# Telehealth provision across allied health professions (AHP): An investigation of reimbursement considerations for its successful implementation in England

**DOI:** 10.1002/hsr2.991

**Published:** 2022-12-13

**Authors:** Nicola Eddison, Carolyn Royse, Aoife Healy, Enza Leone, Nachiappan Chockalingam

**Affiliations:** ^1^ Centre for Biomechanics and Rehabilitation Technologies Staffordshire University Stoke on Trent UK; ^2^ Royal Wolverhampton NHS Trust Wolverhampton UK; ^3^ Dorset County Hospital NHS Foundation Trust, Dorchester Dorset UK

**Keywords:** allied health personnel, health care costs, national health programs, national health service, tariff, telemedicine

## 1 INTRODUCTION

The use of telehealth is not new, however, its recent ubiquity in the National Health Service (NHS) led to the realization that telehealth can offer people a more tailored elective pathway. Resulting in the UK government declaring that digital technology is fundamental to future patient care with a commitment to deliver “at‐scale virtual consultations.”^1^ This ambitious plan requires strategic financial planning.

## 2 BACKGROUND

While telehealth and telemedicine have been growing over the last few decades, issues regarding their regulation and reimbursement have hindered their integration into healthcare systems around the world.^2^, ^3^, ^4^ The response to the Covid‐19 pandemic saw a swift move to digital platforms and virtual consultations for healthcare services, where it was appropriate, to reduce the spread of the disease, release capacity and continue some level of service.

Within the NHS in England, there are 14 professions considered as allied health professions (AHP)^5^: art therapists, dramatherapists, music therapists, podiatrists, dietitians, occupational therapists, operating department practitioners, orthoptists, osteopaths, paramedics, physiotherapists, prosthetists and orthotists, radiographers, and speech and language therapists. There are differences both within the four nations of the United Kingdom and internationally regarding which professions are classified as AHPs.^6^, ^7^ The prevalence of the use of telehealth varies both between and within individual AHP.

Before the pandemic, the expansion of telehealth was underway, as outlined in the 2019 NHS long‐term plan (formerly known as the 10‐year plan).^8^ The plan set out keen ambitions for the NHS to tackle the major issues it is facing and committed to reducing face‐to‐face outpatient consultations by up to a third by 2024.

## 3 THE CONTEXT

The expansion of telehealth within UK NHS AHP services due to the pandemic was accompanied by changes to the national tariff^9^ to move from Payment by Results to block contracts to ensure that services continued to be funded while activity slowed or ceased. In the United Kingdom, the NHS is a publicly funded system, free at the point of need, with groups of general practices coming together to form clinical commissioning groups (CCGs) in each area to commission services. Before 2003, NHS commissioners tended to agree on block contracts with hospitals, meaning the amount of money a hospital received was fixed, regardless of the number of people it treated. These contracts were a fixed sum based largely on historic funding patterns and locally negotiated annual increases. Payment by “Results” was subsequently introduced, which is an approach to paying providers based on the amount of activity undertaken, in accordance with a national tariff *(a set of rules, prices and guidance that governs the payments made by commissioners to secondary healthcare providers for the provision of NHS services)*. To understand the effect of these changes on reimbursement for AHP telehealth consultations because of the pandemic, a Freedom of Information (FOI) request was sent to all CCGs in England in April 2021, to request information on the current tariffs for face to face and telehealth consultations for AHP services (please note that integrated care boards replaced CCGs in the NHS in England from July 1, 2022). An ethics disclaimer form for this study was submitted to the Staffordshire University Ethics Committee. We present a summary of the information received from the FOI and discuss the implications of current reimbursement structures on the successful implementation and planned expansion of telehealth within AHP services in the NHS. Descriptive statistics are used to present the responses to the FOI request.

## 4 THE CURRENT STATE OF PLAY

Responses to the FOI request were received from 63% (67/107) of the total number of CCGs in England:

- 20 were unable to provide telehealth tariff details
- 15 provided telehealth tariff details
- 14 advised tariffs were based on the national tariff (However, AHP consultations are not mandated national tariffs, therefore, the data could not be attained from the National Tariff document).
- 12 were unable to provide telehealth tariff details but stated that the method of delivery was not stipulated, therefore the tariff was the same as for face‐to‐face consultations.
- 6 refused to supply the information requested citing section 43 of the FOI act, which states “*This information is exempt if it constitutes a trade secret or if disclosure would prejudice the commercial interests of any person or body*.*”*

The level of information provided by the 15 CCGs that provided telehealth tariff details varied widely, with some providing tariff details for only one AHP service and others providing details for several AHP services. Responses were received for 8 of the 14 AHP services: dietetics, occupational therapy, orthoptics, physiotherapy, podiatry, prosthetics & orthotics, radiography, and speech and language therapy.

A total of 67 tariff details were provided for both face to face and telehealth consultations, except for 6 of these 67, all the tariffs stated that telehealth consultation tariffs were lower than their face‐to‐face equivalent. On average the telehealth consultation tariffs were 36% lower than face‐to‐face tariffs, and for those that were lower, the differences ranged from −7% to −85%. Variance in the differences were seen across all the AHPs. The tariff which showed the highest difference was for a speech and language consultation (£164.43 face‐to‐face vs. £25.22 telehealth), while the lowest difference was for a physiotherapy consultation (£65.53 face‐to‐face vs. £60.81 telehealth). For the 6 tariffs that were higher for telehealth consultations than for face‐to‐face, the increases ranged from 6% to 70%. The tariff which showed the highest difference was for a physiotherapy consultation (£37.29 face‐to‐face vs. £63.48 telehealth), while the lowest difference was for a podiatry consultation (£36.20 face‐to‐face vs. £38.33 telehealth).

## 5 THE WAY FORWARD

The data shown in the report confirms that there are vast differences across the NHS in England with some CCGs paying the same amount regardless of method of delivery, some paying 6.5 times more for a face‐to‐face consultation, and few paying more for telehealth consultations. Even though national tariff documents from 2017^10^, ^11^ have encouraged providers and commissioners to agree on local prices to further incentivise the increased use of telehealth consultations, suggesting that previous approaches did not provide appropriate incentive to move to alternative care models. The tariff documents themselves leave much for individual CCGs to interpret and do not make any suggestion of levelling up the playing field for tariffs paid for consultations which could be delivered by telehealth. The fact that CCGs continued to pay significantly less in many organizations according to the FOI data suggests that this guidance was not implemented in many cases, at the detriment of alternative delivery of AHP service consultations.

The key factor which might potentially prevent the widespread adoption and implementation of telehealth consultations is the payment structure from CCGs for non‐face‐to‐face consultations. To realize the comprehensive and wide‐ranging digital strategy outlined in the NHS Long Term Plan,^8^ to enable NHS services to work in a novel and digitalized way, and to offer most patients a “digital first” option by 2029, the infrastructure of digitalization in the NHS must be robust. This includes parity of payment between face‐to‐face consultations and telehealth alternatives, alongside a digitally literate workforce and appropriate standardized guidance and training for staff carrying out telehealth consultations.^12^ While we present our findings on telehealth reimbursement from an English perspective, our findings have wider implications, as healthcare systems across the world need to address reimbursement issues to ensure the successful integration of telehealth.

## AUTHOR CONTRIBUTIONS

**Nicola Eddison**: conceptualization; data curation; writing – review & editing. **Carolyn Royse**: conceptualization; data curation; writing—original draft. **Aoife Healy**: data curation; methodology; writing – review & editing. **Enza Leone**: writing – review & editing. **Nachiappan Chockalingam**: conceptualization; funding acquisition; project administration; resources; supervision; writing – review & editing.  **All authors**: read and approved the final version of the manuscript.

## CONFLICTS OF INTEREST

The authors declare no conflicts of interest.

## TRANSPARENCY STATEMENT

The lead author Nachiappan Chockalingam affirms that this manuscript is an honest, accurate, and transparent account of the study being reported; that no important aspects of the study have been omitted; and that any discrepancies from the study as planned (and, if relevant, registered) have been explained.

## Data Availability

The data for this work was obtained through a freedom of information request, with the responses from individual CCGs publicly available. Nachiappan Chockalingam had full access to all of the data in this study and takes complete responsibility for the integrity of the data and the accuracy of the data analysis.
